# The Selenium Paradox: From Evolutionary Redox Chemistry to Medicinal Chemistry

**DOI:** 10.3390/ijms27125404

**Published:** 2026-06-16

**Authors:** Michela Proto, Chiara Giraldi, Claudio Santi

**Affiliations:** Group of Catalysis Synthesis and Organic Green Chemistry, Department of Pharmaceutical Sciences, University of Perugia, 06123 Perugia, Italy; michela.proto@collaboratori.unipg.it (M.P.); chiara.giraldi@studenti.unipg.it (C.G.)

**Keywords:** selenium, redox, evolution, GPx, antioxidant, pro-oxidant

## Abstract

Selenium has played a fundamental role in the evolution of aerobic life, thanks to its unique redox properties and its incorporation into antioxidant selenoproteins such as glutathione peroxidases (GPx). This evolutionary perspective has inspired decades of research aimed at developing small organoselenium compounds as GPx-like antioxidant drugs. However, despite extensive in vitro evidence and numerous publications, no organoselenium antioxidant has been commercialized, and even Ebselen, the most extensively studied selenium-based drug candidate, has repeatedly failed in multiple clinical trials. In this opinion article, we posit the hypothesis that a conceptual bias may underlie a significant proportion of the research conducted to date in this field. The antioxidant activity of GPx is contingent on a highly regulated enzymatic environment that is extremely difficult to reproduce with small synthetic molecules. Consequently, many compounds described as GPx mimetics may behave less like true antioxidants and more like redox-active electrophiles capable of disrupting complex thiol-dependent equilibria. It is recommended that future research should adopt a more holistic approach to the study of selenium pharmacology, moving beyond a reductionist interpretation of GPx-like activity. Instead, the focus should be on the complex network of cellular redox processes and selective redox targeting. It is only through a more profound mechanistic comprehension of selenium chemistry within biological systems that it will be feasible to ascertain whether organoselenium compounds can genuinely establish a presence within the domain of medicinal chemistry, extending beyond their persistent yet predominantly laboratory-restricted achievements. In a similar vein, undertaking a thorough examination of the mechanisms may facilitate a more profound comprehension of the fate of organoselenium compounds in their intricate interaction with biological targets. This, in turn, may enable the conception of novel molecules that function as effective and selective pro-oxidants against specific targets.

## 1. Introduction: Selenium and the Evolution of Aerobic Life

In considering the evolution of life on Earth, the focus is typically placed on large-scale events, including the appearance of oxygen in the atmosphere, the development of multicellular organisms, and the transition of life from the oceans to land. Rarely do we consider the potential impact of trace elements on evolutionary processes. Yet, it can be argued that selenium may have been one of the unheralded protagonists in the emergence of biological complexity.

Recent geochemical evidence suggests that selenium was not only a biochemical tool that enabled advanced life forms to adapt to aerobic conditions, but can also be considered a sensitive indicator capable of providing useful information in the study of one of the most significant periods in Earth’s evolutionary history: the Great Oxidation Event (GOE), which occurred approximately 2.4–2.1 billion years ago. Isotopic traces of selenium, preserved in ancient marine sediments, indicate that surface oxygen levels increased significantly during this period, while the ocean depths remained largely anoxic. As demonstrated by the isotopic distribution of selenium in the sediment, it has also been observed that oxygen levels in the environment subsequently plummeted to very low levels during the subsequent Shunga-Francevillian carbon isotope anomaly. It is hypothesized that these fluctuations in redox potential may have exerted a profound influence on the course of eukaryotic evolution, given the possession of several metabolic processes that are strictly aerobic by eukaryotes [1]. Similarly, the study of selenium isotopes has been utilized also as a tracer of Earth’s redox conditions during the profound changes of the Neoproterozoic (1000–542 million years ago), which revealed an increase in oceanic oxidation and atmospheric oxygen that lasted for a minimum of 100 million years, thereby paving the way for the first animal evolution [2]. Recently, by applying artificial intelligence to large geochemical databases and complex climate models, researchers have analyzed the relationship between oxygen-sensitive metals, organic carbon, and the health of primordial oceans. The results suggest that, contrary to conventional expectations, there was no sudden and complete oxygenation of the deep ocean during the late Neoproterozoic. Rather, there was a gradual and continuous increase in atmospheric oxygen and marine biological productivity over extremely long periods of time [3]. Consequently, the rise of atmospheric oxygen reshaped the course of metabolic evolution. Data from molecular clocks demonstrate that respiratory pathways diversified and spread across various ecosystems during the hundreds of millions of years that followed this atmospheric shift [4].

The data reported by Vadim Gladyshev and coworkers support an evolutionary scenario in which numerous selenoprotein families arose early during eukaryotic evolution and were subsequently shaped by habitat-specific selective pressures. Aquatic environments have been demonstrated to facilitate the retention and diversification of selenium-dependent redox systems. In contrast, terrestrial adaptation has been observed to be accompanied by a contraction or loss of selenoproteomes. Collectively, these observations imply that environmental availability and/or ecological constraints associated with terrestrial life have played a pivotal role in constraining selenium utilization, although the underlying mechanisms remain to be elucidated [5].

This perspective represents a paradigm shift in the way selenium is generally viewed. Selenium should no longer be considered merely a micronutrient with antioxidant properties, but rather a redox modulator acting as a molecular bridge between planetary redox chemistry and biological evolution. The geochemical behavior of selenium has enabled the reconstruction of ancient redox transitions. Similarly, its biological incorporation into living systems may have provided organisms with the biochemical flexibility necessary to survive in increasingly oxygen-rich environments. Alternatively, the amount of available oxygen may have determined the amount of selenium incorporated as a trace element into the protein pool of living species.

The increase in atmospheric oxygen gave rise to both opportunities and dangers. The presence of oxygen has been demonstrated to facilitate more efficient energy production through the process of aerobic metabolism. However, it should be noted that this process also gives rise to the generation of reactive oxygen species (ROS), which have been shown to be capable of causing damage to proteins, lipids, and DNA. This led to a paradoxical situation where organisms were required to first survive in an environment containing oxygen before they could utilize it for their metabolic processes. At this precise juncture, selenium assumes a pivotal role in the evolutionary process, in conjunction with other defense mechanisms against ROS.

## 2. Why Evolution Chose Selenium

The number of selenium atoms incorporated into biological systems is far lower than that of sulfur atoms; estimates suggest approximately one selenium atom for every 10,000–100,000 sulfur atoms. This observation indicates that selenium likely plays a primarily catalytic role in the chemistry of living organisms [6,7].

In contrast to sulfur, to which it is chemically related, selenium exhibits greater polarizability, a property that confers higher nucleophilicity under reducing conditions and higher electrophilicity under oxidizing conditions. This results in greater reactivity and a lower pKa, characteristics that render selenium-containing molecules particularly effective in redox reactions. In biological systems, selenium has been incorporated into selenocysteine, often referred to as the “21st amino acid”, giving rise to selenoproteins specialized in antioxidant defiance and metabolic regulation.

Unlike cysteine, selenocysteine is not a proteinogenic amino acid, a cellular pool of free selenocysteine does not exist, and putative selenocycteinyl-tRNA synthetase never evolved, presumably because of its instability and marked tendency to form the corresponding diselenide. Consequently, evolution selected a highly conserved post-transcriptional incorporation mechanism. The remarkable persistence of this sophisticated machinery throughout evolution is particularly noteworthy. The incorporation of selenocysteine into proteins requires reinterpretation of the UGA stop codon, the presence of SECIS elements (SElenocysteine CInsertion Sequence), specialized tRNAs, and dedicated elongation factors [8,9]. Evolutionary theory generally suggests that biological systems tend toward simplicity and energy efficiency. Consequently, the maintenance of such a complex mechanism strongly suggests that selenium conferred a substantial adaptive advantage. An excellent review article has recently been published on this topic [10]. A few selenoproteins contribute to protecting cells from oxidative stress being part of the overall antioxidant defense system. The best-known examples are glutathione peroxidases, which neutralize hydrogen peroxide and lipid hydroperoxides. Similarly, thioredoxin reductases has a role on maintaining intracellular redox balance [11]. From this perspective, selenium should not be regarded merely as an accessory micronutrient, but rather as a selective evolutionary factor. The transition to aerobic life was determined not only by oxygen availability, but also by the biochemical capacity to manage oxidative damage. Selenium-containing enzymes may therefore have represented a key innovation that enabled organisms first to tolerate, and subsequently to exploit, oxygen-rich environments.

## 3. The Selenium Paradox

It is also important to consider the dual nature of selenium. At physiological concentrations, selenium acts as a protective and regulatory agent; however, at higher concentrations, it becomes toxic and pro-oxidant. The same chemical properties that render selenium highly effective in antioxidant enzymes also explain its toxicity when it is not properly incorporated into protein structures and into biological systems capable of stabilizing and maintaining its reduced form. An excess of selenium has been demonstrated to generate reactive oxygen species, interfere with DNA stability, and induce oxidative damage, particularly due to free radicals. Consequently, life has evolved mechanisms to utilize selenium, as well as systems to strictly regulate it. This delicate balance may provide a rationale for the observed variation in selenium utilization among different species. It has been demonstrated that marine organisms, when exposed to selenium-rich environments (where selenium in various inorganic forms is highly soluble in water), often possess extensive selenoproteomes [12]. In contrast, many terrestrial organisms have been observed to show a reduction or even loss of selenium-dependent proteins. The transition to life on Earth necessitated the adaptation to a less predictable and often scarcer supply of selenium. In order to ensure their survival, terrestrial organisms evolved a process whereby the UGA stop codon sequence of selenocysteine is converted into standard cysteine codons [5]. It is evident that evolution has not universally maximized the use of selenium; on the contrary, organisms have adapted based on the ecological availability of this element and metabolic necessity.

## 4. From Evolutionary Redox Chemistry to Medicinal Chemistry

When analyzing the role of selenium as a redox modulator in biological systems, capable of responding to and perhaps even driving evolutionary pressures, it becomes clear that the design of selenium-containing drugs requires careful consideration. To date, the biological involvement of selenium in antioxidant defense systems has led to extensive speculation regarding its therapeutic potential as an antioxidant.

**Figure 1:**
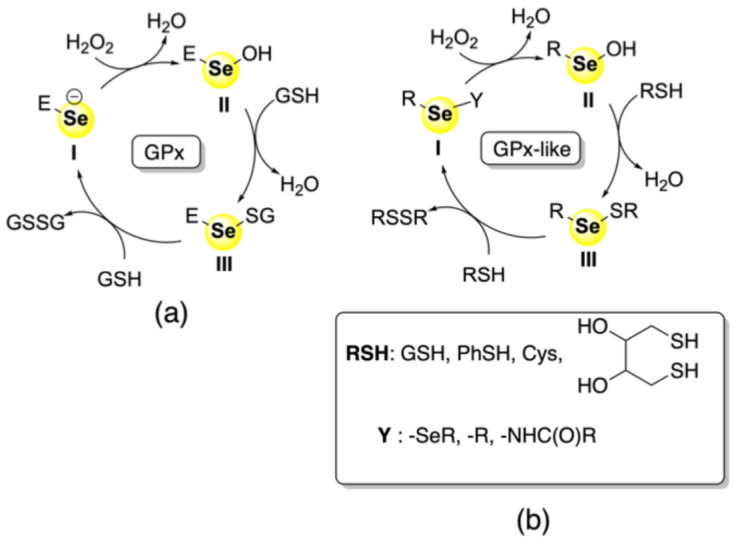
Schematic representation of (**a**) the catalytic mechanism of GPx in comparison with (**b**) a GPx-like cycle reproduced in vitro using selenated small molecules.

For several decades, researchers focused on the synthesis and investigation of small organoselenium molecules displaying apparent antioxidant activity [13,14,15]. Several classes of selenium derivatives were investigated as glutathione peroxidase (GPx) mimetics following the identification of GPx as the first selenoenzyme by Rotruck and Flohè in 1973 [16]. GPx, together with peroxiredoxins and catalase represent the predominant class of enzymes responsible for elimination of ROS in cells. It catalyzes the reduction of harmful peroxides, such as hydrogen peroxide and lipid peroxides, into less toxic derivatives, while simultaneously oxidizing two molecules of reduced glutathione (GSH) into glutathione disulfide (GSSG) (Figure 1a) [17].

These studies contributed significantly to elucidating both the catalytic role of selenium in selenoenzymes and the mechanisms through which different classes of selenium compounds—including diselenides, selenides, and benzoselenazolones—react in the presence of hydrogen peroxide and sulfur-containing cofactors such as GSH, dithiothreitol (DTTred), and thiophenol (PhSH) [18,19,20]. Under these conditions, most selenium derivatives promote peroxide reduction coupled with thiol oxidation. The direct or indirect measurement of the rate of thiol oxidation is often referred to as GPx-like activity. (Figure 1b) [21,22].

Nevertheless, despite the large number of publications claiming antioxidant properties for selenium compounds, concrete therapeutic applications of in vitro GPx mimetics remain unrealized [23]. To date, there is not a single marketed drug containing selenium. Furthermore, the pharmaceutical potential of Ebselen, still considered one of the most promising organoselenium compounds, has been extensively explored yet repeatedly failed to fulfil expectations.

## 5. Why GPx-Mimetics Often Fail (The Nucleophilic Face of Selenium)

The selenium atom in human GPx enzymes participates in a catalytic triad involving neighboring amino acids, such as Sec, Gln, and Trp, which stabilize the unusual selenol oxidation state (-SeH). Moreover, the selenol group possesses a pKa of approximately 5.2, allowing it to exist predominantly in the deprotonated selenolate form (-Se−) at physiological pH [24]. In this form (I, Figure 1), selenium displays strong nucleophilicity and is readily oxidized by peroxides to generate the corresponding selenenic acid (II). During the catalytic cycle, this intermediate is subsequently reduced back to the active selenolate form through oxidation of two GSH molecules to GSSG.

The catalytic triad also plays a crucial role in preventing overoxidation of selenium to seleninic, perseleninic, or selenonic acids, species that are considerably more difficult to reduce back to the catalytically active state. Furthermore, non-bonding interactions within the enzymatic environment facilitate catalyst regeneration by modulating the electrophilicity of the selenium and sulfur atoms in intermediate III. In this intermediate, the sulfur atom must remain more electrophilic than selenium to favor nucleophilic attack by the second GSH molecule. This allows regeneration of the selenolate while avoiding unproductive thiol exchange at selenium that would interrupt the catalytic cycle [25].

It is evident that this finely balanced network of weak interactions is responsible for the precise modulation of selenium redox properties throughout the catalytic cycle. Such conditions are extremely difficult to reproduce in small synthetic organic molecules. For instance, it was reported that GPx1 exhibited a kcat/KM (×10^3^ M^−1^ s^−1^) of 854.2, which is significantly higher than those of Ebselen (3.6), diphenyldiselenide (2.0), and PhSeZnCl (0.4) [26].

This limitation remains one of the major challenges in the design of efficient GPx-like antioxidants and is further complicated by the fact that selenium compounds target not a single receptor, but rather a complex network of redox equilibria capable of triggering unpredictable biological cascades.

## 6. Towards a Targeted Redox Pharmacology: Methodological and Conceptual Limitations (The Electrophilic Face of Selenium)

**Figure 2:**
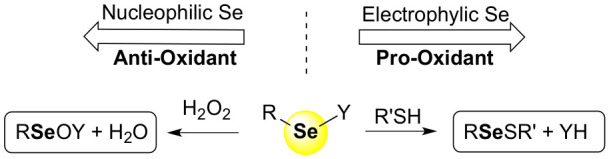
Anti-oxidant vs. Pro-oxidant activity.

An increase in selenium electrophilicity decreases its reducing properties while increasing its susceptibility to nucleophilic attack by free thiols or thiolates, leading to the formation of covalent chalcogen–chalcogen bonds (Figure 2) [27].

In 2012, Lee et al. introduced the term “selenium paradox” to describe the bimodal action of selenium in living systems, whereby both deficiency and excess are associated with severe side effects due to the extremely narrow optimal intake range [28].

The non-specific electrophilic and pro-oxidant activity of many organoselenium compounds is largely responsible for their cytotoxicity. These molecules frequently react with numerous oxidizable cellular targets, particularly free cysteine residues and zinc-finger domains. Conversely, the possibility of directing oxidation selectively toward specific oxidizable targets may represent a more promising strategy for medicinal applications of organoselenium compounds.

**Figure 3:**
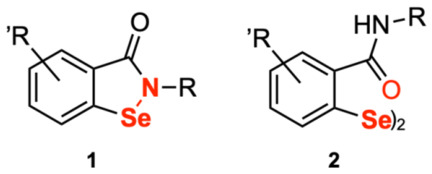
Generic structures for Ebselen-like and DiSeBAs, in red the heteroatoms involved in covalent or non-bonding interactions.

In 2015, we demonstrated that the zinc-finger domain of the HIV retroviral nucleocapsid protein 7 (NCp7) represents a preferential target for a series of 2,2′-diselenobisbenzamides (DISeBAs) (compound **2**, Figure 3) [29]. Inhibition of this protein resulted in broad anti-HIV activity with EC50 values in the low micromolar range and appreciable selectivity indices in both acutely and persistently infected cells, including activity against multidrug-resistant HIV-1 strains and clinical isolates. Similarly, Ebselen (Figure 3, structure **1**; R = Ph and R′ = H) proved to be an effective covalent inhibitor of New Delhi metallo-β-lactamase (NDM-1).

As observed for DISeBAs targeting NCp7, Ebselen interacts with a zinc-finger domain in NDM-1. Tandem mass spectrometry studies demonstrated formation of a covalent Se–S bond involving cysteine 221 within the active site [30].

A comparable inhibitory effect has been observed for the Mpro protein of the SARS-CoV-2 virus, induced by various Ebselen derivatives and their corresponding diselenides [31]. However, the study found that all organoselenium compounds, particularly the more electrophilic ones (such as Ebselen), exhibited strong inhibitory activity against the enzyme. This activity was not always replicated in infected cells. It is logical to assume that neither Ebselen nor the diselenides can reach the target cysteine intact. Especially for Ebselen, high reactivity towards glutathione has been demonstrated, and it is reasonable to assume that the first chemical event involving an organoselenium compound in a biological environment is precisely its reaction with glutathione [32]. This aspect has received scant attention from the research community thus far. However, it is the conviction of the present author that an in-depth study of the formation and reactivity of the selenium-sulfur bond in biological systems is essential for understanding the network of thiol exchange equilibria involving organoselenium compounds. It is only through such rigorous research that we can hope to determine the fate of these compounds and, by extension, their biological activity as well as the mechanism that produces this activity.

These observations suggest that the future therapeutic potential of selenium chemistry lies not so much in the development of generic antioxidants, but rather in the selective targeting of highly reactive protein domains that are sensitive to redox reactions. To achieve this objective, however, it is necessary to conduct further research into the redox chemical mechanisms that govern the fate of each organoselenium compound when it enters a biological system. In this context, selenium should no longer be considered simply as an antioxidant scaffold, but rather as a chemically modulable, redox-active pharmacophore, shaped by billions of years of evolutionary selection. These investigations may also provide insights into the reasons why Ebselen has thus far been unsuccessful in surviving any clinical trial, despite its low toxicity and high activity in all enzymatic tests. In the context of covalent enzyme inhibition, it is imperative to recognize the dynamic nature of the system, wherein the Se-S bond with the catalytically essential cysteine is susceptible to reduction and detoxification by glutathione or other free thiols. It is hypothesized that ebselen is likely to lack a molecular scaffold capable of stabilizing this bond, thereby preventing its reduction (and thus rapid detoxification). It is submitted that, in the event of the catalytic cysteine being considered as the target receptor, an approach to molecular design that closely resembles classical pharmaceutical chemistry would be permitted.

## 7. Is There No Potential for Antioxidant Activity?

The narrative of how selenium was selected by evolution as a pivotal element in the antioxidant defense of living systems and how it was integrated into biological systems offers valuable insights.

(1) The superiority of selenium over sulfur is attributable to its ability to assume a −1 oxidation state, which at physiological pH confers strong electron-withdrawing properties, thereby increasing its reactivity towards peroxides.
(2) This oxidation state is difficult to reproduce in structures not stabilized by intramolecular interactions (and thus outside the protein structure).
(3) The reaction of a selenol with a peroxide generates an electrophilic and pro-oxidant species that performs its antioxidant role thanks to the presence of an excess of glutathione and glutathione reductase, which maintains the reducing potential within the cell.

The nucleophilic form of organoselenium compounds is characterized by extreme instability; consequently, the compounds that can reasonably be utilized as biologically active molecules are either mild electrophiles, such as diselenides, or stronger electrophiles, such as Ebselen. These elements will interact with the complex network of redox equilibria, producing an overall effect that is difficult to predict. It is hypothesized that the development of small organoselenium molecules with antioxidant activity requires a holistic approach, considering biological redox as a complex system in which the compound causes a disturbance that shifts all the equilibria, producing an overall effect that is not simply the sum of the individual effects. It is only by shifting our paradigm and moving from a reductionist to a holistic perspective that we can truly envisage the possibility of developing compounds that act as redox modulators. Such modulators would support cellular defense against oxidative stress and the damage it causes.

Very recent studies demonstrate that a holistic approach integrating omics technologies and artificial intelligence has the potential to lead to the development of new personalized antioxidant therapies, thus opening new avenues for clinical translation and for interventions targeting specific diseases [33].

In addition to conventional covalent drugs that target nucleophilic cysteines, it is becoming evident that the cysteine redoxome can be regarded as a dynamic regulatory level with intriguing therapeutic potential [34].

## 8. Conclusions

The history of selenium in biology demonstrates that its role is far more complex than that of a simple antioxidant element. Evolution selected selenium because of its exceptional redox properties, but at the same time constrained and tightly regulated its use through sophisticated biochemical mechanisms. This evolutionary balance highlights an important lesson for medicinal chemistry: selenium activity cannot be separated from the biological context in which it operates.

In this opinion article, we suggest that a substantial part of the research devoted to GPx mimetics and selenium-based antioxidants may have been influenced by a conceptual bias. The field has often assumed that reproducing the catalytic cycle of GPx with small synthetic organoselenium compounds would be sufficient to obtain effective antioxidant drugs. However, decades of research have shown that the antioxidant activity observed in simplified in vitro systems rarely translates into therapeutic efficacy in vivo. To date, no organoselenium compound has reached the market as an antioxidant drug, and even Ebselen, despite highly promising results across numerous experimental models, has consistently failed to meet expectations in clinical trials.

These observations should not necessarily be interpreted as evidence that selenium chemistry lacks medicinal potential. Rather, they suggest that the biological activity of organoselenium compounds is likely governed by much more complex mechanisms than simple peroxide reduction. The intrinsic electrophilicity of selenium, its rapid interaction with glutathione and cellular thiols, and its ability to perturb interconnected redox equilibria make these compounds global redox modulators rather than straightforward GPx substitutes.

For this reason, we believe that future research should move beyond the traditional reductionist approach centered exclusively on GPx-like activity assays. A deeper understanding of selenium–sulfur exchange reactions, thiol reactivity, and selective targeting of redox-sensitive protein domains may provide a more realistic path toward therapeutic applications. In this context, organoselenium compounds should not be viewed merely as antioxidant mimetics, but as evolutionarily inspired redox-active pharmacophores whose activity emerges from complex interactions within biological systems.

Only by embracing this broader and more holistic perspective will it be possible to establish whether selenium chemistry can truly generate clinically useful molecules and finally bridge the gap between fascinating laboratory results and real therapeutic innovation.

## Data Availability

No new data were created or analyzed in this study. Data sharing is not applicable to this article.
